# Hyperglycemia Impairs the Expression of Mediators of Axonal Regeneration During Diabetic Wound Healing in Rats

**DOI:** 10.3390/biomedicines13122994

**Published:** 2025-12-06

**Authors:** Jaylan Patel, Vy Ho, Tommy Tran, Betelhem Teshome, Vikrant Rai

**Affiliations:** Department of Translational Research, College of Osteopathic Medicine of the Pacific, Western University of Health Sciences, Pomona, CA 91766, USA; jaylan.patel@westernu.edu (J.P.); vy.ho@westernu.edu (V.H.); tommy.tran@westernu.edu (T.T.); bteshome@westernu.edu (B.T.)

**Keywords:** diabetic wound healing, neuroregeneration, activin A, TNFRSF10B, synaptophysin

## Abstract

**Background/Objectives**: Diabetic foot ulcers (DFUs) are one of the most debilitating complications of diabetes mellitus, characterized by impaired wound healing, chronic inflammation, and neuropathy. Peripheral nerve degeneration plays a critical role in delayed healing, but the molecular mediators linking hyperglycemia, neurodegeneration, and impaired DFU repair remain incompletely understood. This study aims to characterize the expression of activin A, which is a key regulator of fibroblast activity and neuronal growth, tumor necrosis factor receptor superfamily member 10B (TNFRSF10B), which mediates inflammatory and apoptotic signaling, and synaptophysin, which serves as a marker of axonal sprouting and synaptic remodeling in diabetic tissues. **Methods**: Skin tissues during wounding and after healing from control and diabetic Sprague–Dawley rats were analyzed using histological staining, immunohistochemistry, and quantitative real-time polymerase chain reactions. Additionally, rat fibroblasts were treated with hyperglycemic medium to evaluate gene and protein expression in vitro. **Results**: Histological analyses revealed impaired healing in diabetic wounds with reduced collagen deposition, loss of adnexal structures, and disorganized tissue architecture. Gene and protein expression of activin A, TNFRSF10B, and synaptophysin were significantly decreased in diabetic healed tissues compared to controls. In vitro, hyperglycemia induced transient upregulation of activin A and TNFRSF10B at 24 h, followed by a decline at 48 and 72 h. **Conclusions**: These findings indicate that hyperglycemia disrupts key mediators of axonal regeneration in DFUs, potentially contributing to impaired neuronal regeneration and delayed healing. Targeting these molecular pathways may offer therapeutic opportunities to enhance wound repair in DFUs.

## 1. Introduction

Diabetes mellitus is a metabolic disorder characterized by chronic hyperglycemia, leading to widespread systemic complications. In the United States, diabetes is a leading cause of morbidity and mortality, and is associated with significant vascular and neuropathic complications [1]. Among these, diabetic foot ulcers (DFUs) represent one of the most debilitating outcomes, often resulting in chronic wounds, infection, recurrence, amputation, and even death [2]. A key underlying factor in DFU pathogenesis is peripheral nerve degeneration (diabetic neuropathy), which reduces protective sensation and prevents patients from detecting minor injuries before they progress to ulcers. Nerve regeneration significantly promotes wound healing by releasing growth factors (nerve growth factor, NGF) and neurotrophic factors (neuropeptides like substance P). NGF, secreted by various skin cells, including keratinocytes, fibroblasts, and mast cells, stimulates keratinocyte proliferation and migration, as well as fibroblast differentiation into myofibroblast, promoting re-epithelialization and granulation tissue formation, respectively. NGF also plays a role in angiogenesis [3]. Substance P, released by nerve fibers in response to injury, triggers a neurogenic inflammatory response by activating immune cells, stimulates the proliferation and migration of fibroblasts and keratinocytes, and influences the activity of matrix metalloproteinases (MMPs), which are involved in ECM remodeling and scar maturation [4]. Therefore, fostering nerve regeneration is a promising strategy for improving the quality and functionality of wound repair, particularly in cases of chronic or impaired healing [5,6]. This suggests that peripheral nerves are crucial for cutaneous wound healing, and the functional and architectural restoration of the skin [4,7]. Importantly, restoring nerve integrity and recovering the skin–nerve interaction promote DFU healing [8]. However, the molecular mechanisms and the mediators involved linking hyperglycemia, neurodegeneration, and impaired healing remain incompletely understood, highlighting the need for further investigation.

Chronic inflammation plays a critical role in impaired wound repair [9,10,11]. Increased expression of inflammatory mediators such as interleukin (IL)-8, IL-1, IL-6, and tumor necrosis factor (TNF)-α sustains a persistent pro-inflammatory environment, preventing normal tissue regeneration and resulting in a chronic nonhealing ulcer. TNF superfamily members are central to these processes, mediating apoptosis, necroptosis, and inflammatory signaling [12]. Notably, TNFRSF10B (Death Receptor 5), a TNF receptor, has been implicated in neurodegeneration through pro-apoptotic signaling and exosome-mediated pathways in DFUs [13]. The upregulation of TNFRSF10B is further associated with increased cytokine release, including that of IL-8, which perpetuates chronic inflammation and contributes to neuronal injury and delayed healing [14]. The effects of TNFRSF10B depend on its interaction with its ligand. Its interaction with its ligand, TRAIL (TNF-Related Apoptosis-Inducing Ligand), can activate inflammatory pathways like the nuclear factor kappa beta (NF-κB) pathway, leading to the release of inflammatory cytokines [15]. However, TNFRSF10B can also trigger apoptosis in inflammatory cells, which can mitigate inflammation [16]. Apoptosis and inflammation negatively regulate axonal regeneration in the central nervous system, while in the peripheral nervous system, the inflammatory response can sometimes be beneficial if tightly controlled, but excessive or prolonged inflammation is detrimental to repair [17,18].

Axonal regeneration is mediated by activin A and synaptophysin. Activin A promotes axonal regeneration by enhancing the intrinsic growth capacity of neurons and aiding in the reconstruction of neural circuits after injury. It achieves this by activating the phosphoinositide 3-kinase (PI3K)/protein kinase B (AKT)/mammalian target of the rapamycin (mTOR) pathway, which inhibits autophagy, and by upregulating factors like synaptogenesis-related factor Sema3A, which helps rebuild connections in the spinal cord [19,20]. Synaptophysin is a crucial protein for neuro-regeneration because its expression indicates the formation of new synaptic connections and the health of regenerating neurons. It is not a direct promoter of axonal regeneration, but its increased transport via synaptophysin-carrying vesicles is a key component of successful regeneration, particularly after a conditioning peripheral lesion. Conversely, elevated levels of synaptophysin alongside other markers in the CNS are linked to axonal injury and degeneration [21].

However, the role of TNFRSF10B, activin A, and synaptophysin in cutaneous healing is not known. Since nerve regeneration is a vital, active component and plays a critical role in cutaneous healing [22], it is important to investigate the role of these mediators during wound healing. This study aims to investigate the differential expression of TNFRSF10B, activin A, and synaptophysin in control nondiabetic and diabetic skin tissues, before and after wound healing, collected from the Sprague–Dawley rat model of diabetes with cutaneous wound healing. We aim to observe the effects of hyperglycemia on mediators of nerve regeneration during wound healing.

## 2. Materials and Methods

### 2.1. Tissue Collection and Processing

The tissues collected from ongoing studies in the lab were used for this study (R24IACUC013, 13 May 2024). Briefly, tissues were collected from Sprague–Dawley rats, both male and female, aged 6–8 weeks and weighing approximately 180 g, obtained from Charles River Laboratories (Thousand Oaks, CA, USA). The animals were housed in the Animal Resource Facility at the Western University of Health Sciences, Pomona, CA, under a temperature of 22 °C and a 12 h light/dark cycle. The tissues were collected from nondiabetic (ND) control and diabetic (D) rats, during wounding (hereafter control skin) and after healing (healed skin). In this study, we used a total of 28 already collected tissues from seven rats in each condition. The control and healed tissues were collected from dorsum skin during wounding and the healed skin (filling the wound gap) after the wound was healed (not the adjacent tissue or wound bed). The healed skin from nondiabetic rats was collected at day 14 and from diabetic rats at day 21. The control skin from ND and D rats during wounding was collected at day 0. Based on the power analysis using G*power3.0.10 software with an α value of 0.05, the sample size necessary to have at least 90% power to detect a significant change is seven in each group. The wounds were created on dorsum equidistance from the vertebral column on each side using a biopsy punch with a 6 mm diameter and 2 mm depth after removal of the hairs and sterilization of the skin under isoflurane anesthesia. The control groups were fed a normal diet (ND; 20% protein, 70% carbohydrate, and 10% fat; D12450B, Research Diet Inc. New Brunswick, NJ, USA), while the diabetic groups received a high-fat diet (HFD; 35% carbohydrate, 20% protein, and 45% fat; 5.7 kcal/g total; D12451, Research Diet Inc. New Brunswick, NJ, USA), with water ad libitum. Diabetes was induced using a low dose of streptozotocin (STZ; 25 mg/kg, dissolved in 0.1 M sodium citrate buffer, pH 4.4; Sigma-Aldrich, St. Louis, MO, USA) injected intraperitoneally (IP) after six weeks on a high-fat diet (as discussed in [23]). The tissues collected in 10% formalin were processed using LEICA ASP6025 Tissue Processor (Nussloch, Germany), embedded in paraffin, and sectioned using a Leica microtome (Wetzlar, Germany). The sectioned slides were heated at 60 °C for 1 h.

### 2.2. Hematoxylin and Eosin Staining

H&E staining was carried out using the standard procedure established in our laboratory. The slides were first deparaffinized and rehydrated through sequential washes with xylene, graded ethanol solutions (100%, 95%, 80%, and 70%), and distilled water. Tissue sections were then immersed in hematoxylin (Epredia Gill Hematoxylin, # 22-050-202, Fisher Scientific, Hampton, NH, USA) for 45 s, washed in running water, followed by counterstaining with eosin (eight to ten dips, Epredia Shandon Eosin-Y Stain # 6766008, Fisher Scientific, Hampton, NH, USA). After staining, the slides were mounted with Cytoseal 60 (#23-244257, Fisher Scientific, Hampton, NH, USA) using glass coverslips. Images were acquired using a Leica DM6 light microscope (Wetzlar, Germany) at 5×, 10×, and 20×.

### 2.3. Trichrome Staining

Trichrome staining was performed using Stat lab Trichrome kit Masson’s (SKU: KTMTRPT EA, McKinney, TX, USA) by first deparaffinizing and rehydrating the slides through sequential washes in xylene, graded ethanol solutions, and distilled water. The sections were rinsed in water and then re-fixed in Bouin’s solution for 1 h at 56 °C. After washing under running water, the slides were stained with Weigert’s iron hematoxylin working solution for 10 min, followed by Biebrich scarlet–acid fuchsin solution for 6 min. The sections were then washed and immersed in phosphomolybdic–phosphotungstic acid solution for 10 min before transfer to aniline blue solution for 4–5 min. This was followed by two immersions in 1% acetic acid solution for 2 min each. After a final wash in distilled water, the slides were dehydrated rapidly, air-dried, and mounted with CytoSeal 60. Images were acquired using a Leica DM6 light microscope at 5×, 10×, and 20×. Trichrome staining slides were semi-quantitatively analyzed using a scale of 0 to 5, where 0 = minimal blue staining, 1 = very weak blue staining, 2 = weak blue staining, 3 = moderate blue staining, 4 = strong blue staining, and 5 = very strong blue staining. We used tissues from seven rats in each group, and three sections were stained for each rat, and three to five images from each tissue were used for semi-quantitative analysis.

### 2.4. Immunostaining

Immunostaining was performed using the peroxidase–anti-peroxidase method with a horseradish peroxidase-conjugated secondary antibody. Paraffin-embedded sections were deparaffinized, rehydrated, and subjected to antigen retrieval in 1% citrate buffer (Sigma Aldrich, C9999). Following retrieval, tissues were encircled with a Pep Pen and treated with 3% hydrogen peroxide (Sigma Aldrich, H1009) for 15 min to quench endogenous peroxidase activity, then rinsed three times with phosphate-buffered saline (PBS) (5 min each). Non-specific binding was blocked with the blocking solution supplied in the Vectastain Elite ABC kit (Vector Labs, Newark, CA, USA) for 1 h at room temperature. Sections were then incubated overnight at 4 °C with primary antibodies (anti-rat activin A beta A subunit, AF338; TNFRSF10B antibody, ABIN5518794; synaptophysin monoclonal antibody, MA5-14532). After washing (3 × 5 min in PBS), tissues were incubated for 1 h at room temperature with a secondary antibody, followed by washing with PBS and a 30 min incubation with the Vectastain ABC horseradish peroxidase complex. Staining was developed with AEC (3-amino-9-ethylcarbazole) substrate for 2–5 min until a brown-red color appeared. Sections were rinsed with water, counterstained briefly (5–10 s) with hematoxylin, washed under running tap water for 5 min, and mounted with CytoSeal 60. Images were captured with a Leica DM6 microscope at a 100 μm scale. High-magnification images were analyzed using NIH Fiji ImageJ to quantify average staining intensity and area. For each tissue, three sections were processed, and three to four images per section were assessed for statistical analysis.

### 2.5. Quantitative Real-Time Polymerase Chain Reaction

Total RNA was isolated using TRIZOL reagent (Sigma, T9424, St. Louis, MO, USA) according to the manufacturer’s instructions, and RNA concentration was determined using a Nanodrop 2000 spectrophotometer. cDNA synthesis was carried out using the iScript cDNA kit (BioRad, #1708891, Hercules, CA, USA). Quantitative real-time PCR was performed in triplicate using SYBR green dye on a CFX96 system (BioRad Laboratories, Hercules, CA, USA). Primer sequences (Table 1) were purchased from Integrated DNA Technologies (Coralville, IA, USA). The cycling protocol consisted of an initial denaturation step at 95 °C for 10 min, followed by 40 cycles of 95 °C for 30 s, annealing at 55–60 °C (primer-specific) for 30 s, and extension at 72 °C for 30 s. Gene expression was normalized to 18S, and fold changes relative to the control were calculated using the 2^−ΔΔCT^ method. Experiments were performed in triplicate using three biological replicates (*n* = 3). RT-qPCR was used to evaluate the expression of activin A, TNFRSF10B, synaptophysin, and housekeeping gene 18S.

### 2.6. Cell Culture and In Vitro Studies

Rat fibroblasts were cultured in a T75 culture flask until 90% confluence using complete Dulbecco’s Modified Eagle’s Medium (DMEM, ATCC # 30-2002, Manassas, VA, USA) with 10% fetal bovine serum and 1% penicillin streptomycin in a humidified incubator with 5% CO_2_ at 37 °C. The cells were trypsinized for 2–3 min in an incubator and plated in a six-well plate and chamber slides. For PCR, 1 × 10^6^ cells were plated in each well of six-well plate, and 8 × 10^3^ cells were plated in each chamber of the chamber slide for immunofluorescence. The cells in a six-well plate were cultured overnight and then treated with hyperglycemic medium (DMEM with 9.0 g/L glucose). The cells for RNA extraction were collected at 24 h, 48 h, and 72 h. The cells in chamber slides were treated with hyperglycemic medium for 24 h. Total RNA was extracted using TRIZOL, cDNA was prepared, and RT-qPCR was conducted as described above. For immunofluorescence, the cells in the chamber slide were washed with sterile PBS after 24 h, fixed with 10% Formaldehyde, and incubated in 0.01% Triton for 10 min. This was followed by washing with PBS and incubation with blocking solution for one hour. After tipping off the blocking solution, cells were incubated with activin A and TNFRSF10B overnight at 4 °C followed by washing with PBS and incubation with Alexa Fluor 594 for 30 min. The cells were washed, counterstained with DAPI, and scanned using a Leica Fluorescence microscope. The Mean fluorescence intensity was analyzed using ImageJ software.

### 2.7. Statistical Analysis

All data are presented as mean ± SD. The comparison between the two groups was conducted using Student’s *t*-test for statistical significance. The data was used to plot the graphs using GraphPad Prism 10 with nonparametric data with Student’s *t*-test as an option for statistical analysis. A probability value of <0.05 was considered significant. * *p* < 0.05, ** *p* < 0.01, *** *p* < 0.001, and **** *p* < 0.0001.

## 3. Results

### 3.1. Hematoxylin and Eosin Staining Showed Healing with Scar

**Figure 1:**
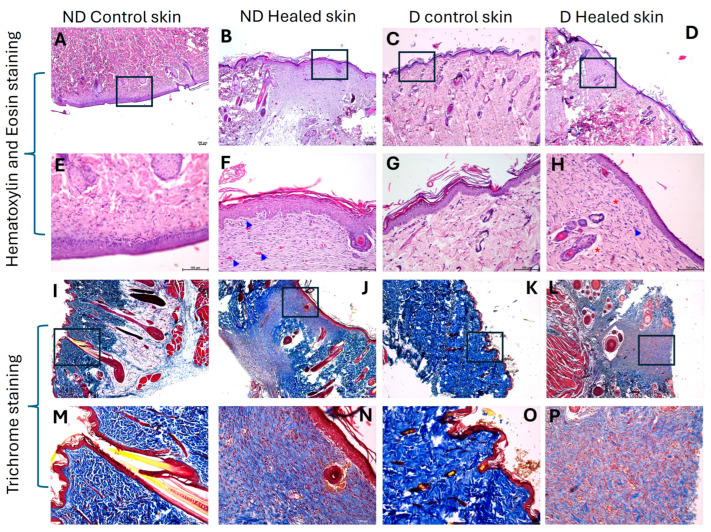
H&E and Trichome staining in the control and diabetic control, and healed tissues. Panels (**A**–**D**,**I**–**L**) are images scanned at 5×, and panels (**E**–**H**,**M**–**P**) were scanned at 20×. The squares in 5× panels represent the area scanned at 20×. The blue arrowheads show blood vessels, and the red asterisks show the presence of minimal fibrosis. ND refers to nondiabetic rats, control before refers to the tissues collected during wounding, healed skin refers to tissues collected after wound was healed, and D refers to diabetic rats. Likewise for diabetic tissues. All images were scanned at a scale of 100 μm.

H&E staining revealed wound healing with scar tissue formation in both nondiabetic (control) and diabetic (D) rats (Figure 1 panels B and D). The H&E staining showed the distorted architecture of the healed tissues. There was no sign of inflammation, and the healed area was devoid of sweat glands, sebaceous glands, and hair follicles. Post-healing samples in diabetic rats demonstrated regions of minimal fibrosis and decreased vessels compared to control healed and control skin (Figure 1).

### 3.2. Trichrome Staining Revealed Decreased Collagen in Diabetic Healed Tissue

Trichrome staining revealed decreased collagen (blue color) in the diabetic healed skin compared to the nondiabetic (ND) control skin, ND healed skin, and diabetic (D) control skin (Figure 1 panels I–P). On a scale of 0–5 with 0 as no color and 5 as a deep blue color, diabetic healed skin showed 1, ND control skin and diabetic control skin showed 2–3, and ND control healed skin showed 1–2, suggesting decreased collagens in diabetic healed skin (Figure 1 panels I–P).

### 3.3. Real-Time Quantitative Polymerase Chain Reaction (RT-qPCR)

**Figure 2:**
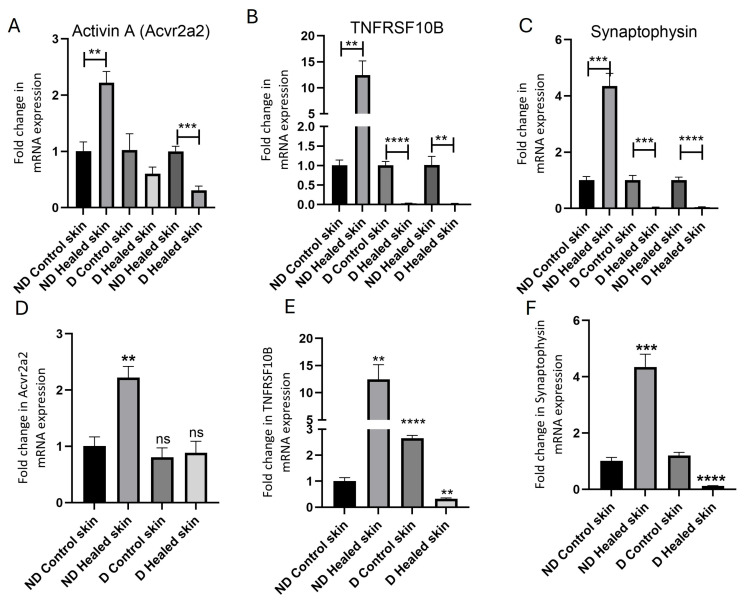
Real-time quantitative polymerase chain reaction (RT-qPCR) analysis of mRNA expression levels of activin A (**A**,**D**), TNFRSF10B (**B**,**E**), and synaptophysin (**C**,**F**). In panels (**A**–**C**), the comparison for the folds change in mRNA expression was performed between the ND control and healed tissues, the diabetic (**D**) control and healed tissues, and the ND healed and D healed tissues. For panels (**D**–**F**), the comparison was performed to compare the fold change in mRNA expression in all other tissues (nondiabetic (ND), diabetic control, and diabetic healed) with the ND control tissues. The data used to plot panels (**A**–**C**) was used to plot panels (**D**–**F**). The asterisks in (**D**–**F**) indicate significance compared to the control skin in nondiabetic rats. Data are presented as mean ± SD. ** *p* < 0.01, *** *p* < 0.001, and **** *p* < 0.0001. ns = not significant.

Within the group (ND control skin vs. ND healed skin, and diabetic control skin vs. diabetic healed skin), comparison of the fold change in mRNA expression showed the following results. In ND control tissue, activin A expression was significantly higher in healed skin compared to control skin (*p* = 0.00119, Figure 2 panel A, first two bars). In contrast, no significant difference was observed between the diabetic control skin and diabetic healed skin (*p* = 0.0777, Figure 2 panel A, third and fourth bars). Between the ND control and diabetic healed tissues, activin A expression was significantly greater in the ND control compared to diabetic tissue (*p* = 0.000509, Figure 2 panel A, last two bars). TNFRSF10B expression was significantly elevated in the ND healed tissues compared to ND control skin (*p* = 0.00191, Figure 2 panel B, first two bars). In the diabetic tissues, TNFRSF10B expression was significantly reduced in the diabetic healed tissues compared to diabetic control tissues (*p* = 0.0000693, Figure 2 panel B, third and fourth bars). Between the ND healed and diabetic healed groups, TNFRSF10B expression remained significantly higher in the ND healed compared to diabetic healed tissue (*p* = 0.00153, Figure 2 panel B, last two bars). Synaptophysin expression was significantly higher in the ND healed compared to control tissue (*p* = 0.000255, Figure 2 panel C, first two bars). In the diabetic tissues, synaptophysin expression was significantly reduced in the healed tissues compared to control tissues (*p* = 0.000508, Figure 2 panel C, third and fourth bars). Between the ND healed and diabetic healed groups, synaptophysin levels were significantly higher in the ND healed compared to diabetic healed tissue (*p* = 0.0000965, Figure 2 panel C, last two bars).

Comparison of all tissues compared to the ND control skin tissues showed a significant increase in gene expression of activin A in the ND healed tissues, but no significant change in diabetic control and the diabetic healed tissues compared to ND control tissues (Figure 2 panel D). The gene expression of TNFRSF10B significantly increased in the ND healed tissues and diabetic control tissues, and significantly decreased in the diabetic healed tissues compared to control tissues (Figure 2 panel E). The gene expression of synaptophysin was significantly increased in the ND healed tissues and significantly decreased in the diabetic healed tissues compared to ND control tissues (Figure 2 panel F). There was no significant change in the diabetic control tissues compared to ND control tissues.

### 3.4. Immunohistochemistry

**Figure 3:**
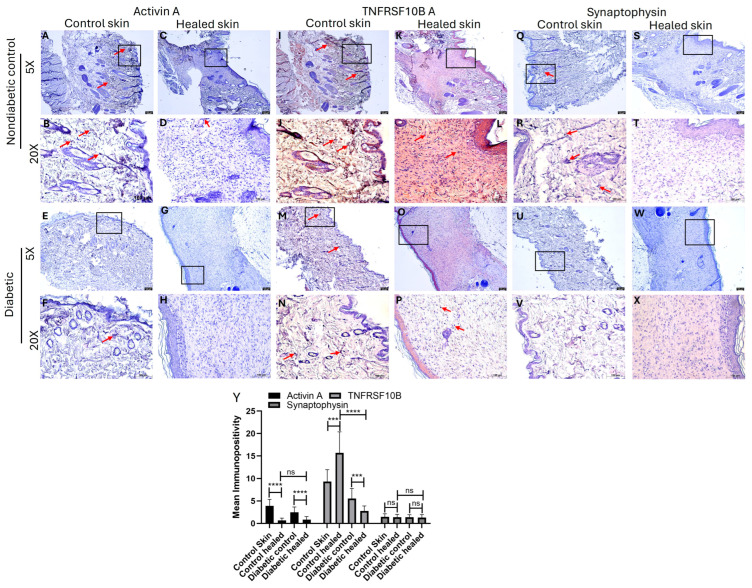
Immunohistochemistry staining of control (skin collected during wounding) and healed (skin collected after wound healed) skin in the nondiabetic (ND) control and diabetic (D) rats. Panels (**A**–**D**,**I**–**L**,**Q**–**T**) (nondiabetic control rats) and panels (**E**–**H**,**M**–**P**,**U**–**X**) (diabetic rats). Activin A (panels **A**–**H**), TNFRSF10B (panels **I**–**P**), and synaptophysin (panels **Q**–**X**). Control skin was collected during wounding (panels **A**,**B**,**E**,**F**,**I**,**J**,**M**,**N**,**Q**,**R**,**U**,**V**) and healed skin was collected during sacrifice after the wound healed (panels (**C**,**D**,**G**,**H**,**K**,**L**,**O**,**P**,**S**,**T**,**W**,**X**). The squares/rectangles in 5× show the area scanned at 20×. The red arrows point out the positive immunostaining/expression of the corresponding protein. Panel (**Y**) shows the average immunopositivity. *** *p* < 0.001, and **** *p* < 0.0001. ns = not significant.

Immunohistochemistry (IHC) revealed significantly decreased expressions (immunopositivity, shown with red arrows) of activin A in the diabetic control and diabetic healed tissues (Figure 3, panels E–H) compared to the ND control and ND healed skin (Figure 3, panels A–D and panel Y). The protein expression of TNFRSF10B (DR5) was higher in the healed control (Figure 3I,J) compared to the ND control (Figure 3, panels K,L,Y). The protein expression of TNFRSF10B decreased in the diabetic control and diabetic healed compared to the ND control and ND healed. The protein expression of TNFRSF10B was significantly decreased in the diabetic healed compared to diabetic controls (Figure 3, panel Y). The protein expression of synaptophysin in the ND control and ND healed skin was minimal (Figure 3, panels Q–T), and slightly decreased in the diabetic control and diabetic healed skin (Figure 3, panels U–X) compared to the ND control skin. There was no significance in the protein expression of synaptophysin between groups (Figure 3, panel Y). While comparing the protein expression of activin A and synaptophysin, the protein expression of activin A was higher than synaptophysin in the control and healed tissues in both ND and diabetic rats. It was noted that minimal immunopositivity of these mediators was masked by hematoxylin, and to show immunopositivity (brown color) we have included the images after color deconvulation (Supplementary Figure S1).

### 3.5. In Vitro Studies Revealed Effects of Hyperglycemia on the Expression of Activin A and TNFRSF10B

**Figure 4:**
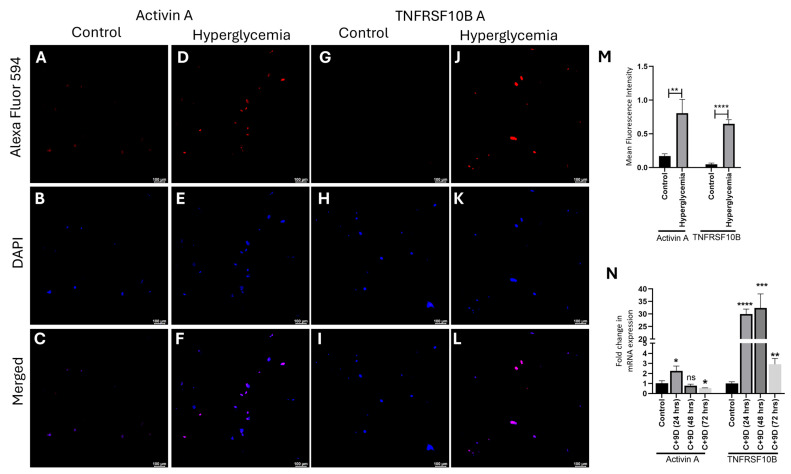
Immunofluorescence staining for protein expression (panels **A**–**L**), mean fluorescence intensity for activin A and TNFRSF10B (panel **M**), and polymerase chain reaction (panel **N**) for gene expression of activin A and TNFRSF10B. All images were scanned at 10μM. The RT-qPCR data are presented as mean ± SD. The probability value of *p* < 0.05 was considered significant. * *p* < 0.05, ** *p* < 0.01, *** *p* < 0.001, and **** *p* < 0.0001. C+9D- control medium with 9 mg/dL glucose (hyperglycemic medium). The asterisks indicate significance compared to control cells for each gene. ns = not significant.

Immunofluorescence staining of the fibroblasts treated with hyperglycemia showed increased fluorescence intensity compared to cells cultured in normal medium for both activin A (Figure 4, panels C and F) and TNFRSF10B (Figure 4, panels I and L) at 24 hrs (Figure 4, panel M). We did not stain for synaptophysin because of its absence in fibroblasts. The mRNA expression of activin A and TNFRSF10B was found to be significantly increased in cells treated with hyperglycemic medium compared to the control cells (Figure 4, panel N). However, this expression decreased at 48 and 72 h for activin A, remained elevated for TNFRSF10B at 48 h, but significantly decreased at 72 h.

## 4. Discussion

In this study, we found decreased gene and protein expressions of activin A, synaptophysin, and TNFRSF10B in diabetic skin and diabetic healed skin after wounding compared to nondiabetic control skin and healed skin. Activin A is needed for wound healing, as it promotes fibroblast proliferation, migration, and extracellular matrix deposition, which are essential for tissue repair. However, excessive activin A can lead to problematic scarring and fibrosis, so its activity must be carefully balanced [24,25]. On the other hand, activin A is also crucial for nerve regeneration in both the central and peripheral nervous systems, and promotes recovery from nerve damage by reducing inflammation, fostering cell proliferation, and enhancing neurogenesis [20,26]. Neuroregeneration is important for wound repair. Our findings revealed the decreased expression of activin A in diabetic skin, where the wound healing was delayed (21 days in diabetics compared to 14 days in controls). This decreased expression of activin A may be one of the factors contributing to delayed healing in diabetics. However, the literature suggests that hyperglycemia is associated with increased levels of activin A, a protective counter-mechanism to cell damage, and has tissue-specific complex effects [27,28]. Its increased expression with hyperglycemia is supported by our in vitro results. The decreased expression of activin A in diabetic tissues may be due to ongoing fibrosis, and the decreased number of fibroblasts and neuronal cells in skin [26], and these decreased levels may contribute to delayed wound healing. It should be noted that the role of activin A is context dependent. In zebrafish fin regeneration and axolotl limb/Xenopus tail regeneration, activin A signaling (specifically activin-βA) is required for the regeneration process to occur normally. The inhibition of activin signaling in these models leads to a block or impairment of regeneration, including problems with wound healing, cell migration, and blastemal proliferation (17683938). In mammalian skin wound healing, the role is complex: activin A is strongly expressed in wounded skin and promotes keratinocyte proliferation and migration, which aids in normal wound healing. However, in adult mammals, this process leads to scar formation, whereas fetal skin (which has different expression profiles of TGF-β family members) heals without scarring [29,30]. Thus, a decrease in activin A during wound healing will negatively affect wound healing and prolong the healing process. However, the role of activin A after muscle injury is different. In mice with acute muscle injury, local activin A levels increase and contribute to muscle degeneration and a sustained inflammatory response. Neutralizing activin A with a specific antibody accelerates muscle repair and functional recovery by promoting a more efficient clearance of damaged tissue and enhancing the activation of muscle precursor cells [31].

Synaptophysin is another factor crucial for nerve regeneration, particularly in the stabilization of newly formed synaptic connections, and is an indicator of nerve regeneration during wound healing. It is a reliable marker for monitoring the extent of axonal sprouting and synaptic terminal growth after injury [32,33]. Synaptophysin expression has also been related to cutaneous wound healing [34]. However, no study has investigated the expression of synaptophysin in diabetic wound healing. Our study revealed the significantly decreased protein and gene expressions of synaptophysin in diabetic tissues compared to control tissues. A decreased synaptophysin expression in the diabetic tissues suggests decreased axonal regeneration in the healing tissues. A decreased axonal/nerve regeneration may contribute to delayed healing in a diabetic wound. A decreased expression of synaptophysin in diabetic tissues is supported by the fact that hyperglycemia decreases synaptophysin expression [35]. Synaptophysin is an integral membrane protein of synaptic vesicles, remains attached to the membrane during the process of exocytosis, and is not released into the extracellular space. Ongoing fibrosis with decreased nerves in the healed wound may be another reason for decreased synaptophysin expression in both the control and diabetic healed wounds compared to their respective control. Decreased synaptophysin expression is associated with impaired neural regeneration and recovery of function in animal models. Synaptophysin is a key protein involved in the formation and plasticity of synapses, and its reduction negatively impacts the ability of neurons to form new, functional connections after injury or in neurodegenerative conditions [36,37,38]. The results of these studies have suggested that lower levels of synaptophysin result in fewer functional synaptic connections, a process crucial for nerve regeneration, the recovery of function after injury, and less efficient recycling and endocytosis of synaptic vesicles during sustained neuronal activity, leading to a faster depletion of the pool of available synaptic vesicles and thus impaired neurotransmission.

TNFRSF10 (Death Receptor 5) has a complex and multifaceted role in wound healing that depends on the cell type and stage of tissue repair. While it is best known for inducing apoptosis in damaged cells, it also plays non-apoptotic, pro-survival, and anti-fibrotic roles. In the initial phases of tissue repair, such as in naive or resting fibroblasts, TNFRSF10B promotes proliferation and survival rather than cell death. In later stages, after fibroblasts have transformed into myofibroblasts and laid down the initial extracellular matrix, TNFRSF10B switches to a pro-apoptotic function [39]. It should also be noted that TNFRSF10B function depends on its interaction with its ligand and can promote inflammation by activating the NF-κB pathway [15], or may trigger apoptosis in inflammatory cells, which can mitigate inflammation [16]. We found a decreased expression in the diabetic control and healed skin compared to the nondiabetic control and healed skin. A decreased expression of TNFRSF10B in diabetic skin may be due to either an irregular immune response or altered apoptosis, contributing to delayed healing, which needs to be further investigated. Our in vitro studies revealed increased gene and protein expression with hyperglycemia, and these findings are in line with previous reports of increased TNFRSF10B expression with hyperglycemia [40]. However, a reduced expression in diabetic skin tissues raises concerns, and this may be due to a decreased number of fibroblasts, which express TNFRSF10B, due to ongoing fibrosis in the healing tissues. The expression of TNFRSF10B changes with changing fibroblast phenotype [41]. TNFRSF10B is not directly related to nerve regeneration but may play a role through apoptosis and warrants further research.

## 5. Conclusions

The clinical implications of diabetic foot ulcers are well recognized, but the molecular mechanisms underlying their impaired wound healing are not fully understood. This study demonstrates the decreased expressions of activin A, TNFRSF10B, and synaptophysin in diabetic tissues compared with controls. These findings may support the notion that disrupted nerve regeneration contributes to delayed wound healing in diabetes. By highlighting a potential mechanistic link between hyperglycemia, altered neuronal remodeling, and impaired wound repair, these findings establish a basis for further investigation into the signaling pathways involved. Future studies with larger sample sizes are warranted to validate these results and explore the therapeutic potential of targeting these mediators to enhance DFU healing.

**Limitations of the study and future directions:** This study highlights the effects of hyperglycemia on the gene and protein expression of the mediators of axonal regeneration in association with wound healing. The study also highlights that activin A, synaptophysin, and TNFRSF10B may be therapeutic targets to promote wound healing in chronic DFUs. Despite these findings, there are a few limitations. This study used fibroblasts for in vitro studies to investigate the effects of hyperglycemia. These mediators of axonal regeneration are normally not expressed on fibroblasts but are expressed with stimulation. To confirm the effects of hyperglycemia, cells specifically expressing these mediators should be used in future studies. Next, the tissues should be stained for neurons/axons to evaluate the effects of hyperglycemia on their density in the tissues. Furthermore, neuronal cells may be used in vitro to analyze the effects of hyperglycemia. As mentioned above, we tried to culture neuronal cells, but they never reached the confluence of 30–40% in 14 days (a limitation of not using these cells in this study). Further, knock-in and knock-down studies should be conducted to establish the role of these mediators in wound healing, both in control and hyperglycemic conditions. The study has not discussed the details of diabetes induction and the healing timeline. These aspects have been submitted in another manuscript and will be published soon.

## Figures and Tables

**Table 1:** Forward and reverse primer sequences used in RT-qPCR.

Gene	Primer Sequence
Activin A	Forward: 5′-GGGGATTGTCATTTGTGCGT-3′ Reverse: 5′-GTGGTCCAGGGTCCTGAGTA-3′
TNFRSF10B	Forward: 5′-CCAGGATAGCCTACAACTCAAG-3′ Reverse: 5′-AGGAGAGAGAGAGAGAGAGAGA-3′
Synaptophysin	Forward: 5′-ACTTTCTCTCCTTCCTCCTCTC-3′ Reverse: 5′-TCCACTCAGTCTACTGCTCTAC-3′
18S	Forward: 5′-GTAACCCGTTGAACCCCATT-3′ Reverse: 5′-CCATCCAATCGGTAGTAGCG-3′

## Data Availability

All data related to this manuscript is included.

## Supplementary Materials

The following supporting information can be downloaded at: https://www.mdpi.com/article/10.3390/biomedicines13122994/s1. Figure S1: Deconvulated images to show immunopositivity in different groups.

## Abbreviations

The following abbreviations are used in this manuscript:

CNA	Central nervous system
DFU	Diabetic foot ulcer
DR5	Death Receptor 5
ECM	Extracellular matrix
IL	Interleukin
IHC	Immunohistochemistry
MMPs	Matrix metalloproteinases
mTOR	Mammalian target of rapamycin
NFG	Nerve growth factor
NF-κB	Nuclear factor kappa beta
PI3K	Phosphoinositide 3-kinase
TNF-α	Tumor necrosis factor-alpha
TRAIL	TNF-Related Apoptosis-Inducing Ligand
TNFRSF10B	Tumor Necrosis Factor Receptor Superfamily, Member 10B

## References

[1] Sapra A., Bhandari P., Wilhite Hughes A. (2025). Diabetes (nursing).

[2] McDermott K., Fang M., Boulton A.J.M., Selvin E., Hicks C.W. (2023). Etiology, Epidemiology, and Disparities in the Burden of Diabetic Foot Ulcers. Diabetes Care.

[3] Liu Z., Wu H., Huang S. (2021). Role of NGF and its receptors in wound healing (Review). Exp. Ther. Med..

[4] Xing L., Chen B., Qin Y., Li X., Zhou S., Yuan K., Zhao R., Qin D. (2024). The role of neuropeptides in cutaneous wound healing: A focus on mechanisms and neuropeptide-derived treatments. Front. Bioeng. Biotechnol..

[5] Kawamoto K., Matsuda H. (2004). Nerve growth factor and wound healing. Prog. Brain Res..

[6] Wang K., Song B., Zhu Y., Dang J., Wang T., Song Y., Shi Y., You S., Li S., Yu Z. (2024). Peripheral nerve-derived CSF1 induces BMP2 expression in macrophages to promote nerve regeneration and wound healing. NPJ Regen. Med..

[7] Emmerson E. (2017). Efficient Healing Takes Some Nerve: Electrical Stimulation Enhances Innervation in Cutaneous Human Wounds. J. Investig. Dermatol..

[8] Ji X., Zhou J., Zhou Z., Liu Z., Yan L., Li Y., Guo H., Su W., Wang H., Ni D. (2024). Recovering skin-nerve interaction by nanoscale metal-organic framework for diabetic ulcers healing. Bioact. Mater..

[9] Littig J.P.B., Moellmer R., Estes A.M., Agrawal D.K., Rai V. (2022). Increased Population of CD40+ Fibroblasts Is Associated with Impaired Wound Healing and Chronic Inflammation in Diabetic Foot Ulcers. J. Clin. Med..

[10] Rai V., Moellmer R., Agrawal D.K. (2022). The role of CXCL8 in chronic nonhealing diabetic foot ulcers and phenotypic changes in fibroblasts: A molecular perspective. Mol. Biol. Rep..

[11] Shofler D., Rai V., Mansager S., Cramer K., Agrawal D.K. (2021). Impact of resolvin mediators in the immunopathology of diabetes and wound healing. Expert Rev. Clin. Immunol..

[12] Kearney C.J., Cullen S.P., Tynan G.A., Henry C.M., Clancy D., Lavelle E.C., Martin S.J. (2015). Necroptosis suppresses inflammation via termination of TNF- or LPS-induced cytokine and chemokine production. Cell Death Differ..

[13] Dai M., Yan L., Yu H., Chen C., Xie Y. (2023). TNFRSF10B is involved in motor dysfunction in Parkinson’s disease by regulating exosomal alpha-synuclein secretion from microglia. J. Chem. Neuroanat..

[14] O’Hara A.M., Bhattacharyya A., Bai J., Mifflin R.C., Ernst P.B., Mitra S., Crowe S.E. (2009). Tumor necrosis factor (TNF)-alpha-induced IL-8 expression in gastric epithelial cells: Role of reactive oxygen species and AP endonuclease-1/redox factor (Ref)-1. Cytokine.

[15] Tang W., Wang W., Zhang Y., Liu S., Liu Y., Zheng D. (2009). TRAIL receptor mediates inflammatory cytokine release in an NF-kappaB-dependent manner. Cell Res..

[16] Qiao X., Guo S., Meng Z., Gan H., Wu Z., Sun Y., Liu S., Dou G., Gu R. (2025). Advances in the study of death receptor 5. Front. Pharmacol..

[17] Yiu G., He Z. (2006). Glial inhibition of CNS axon regeneration. Nat. Rev. Neurosci..

[18] Gu D., Xia Y., Ding Z., Qian J., Gu X., Bai H., Jiang M., Yao D. (2024). Inflammation in the Peripheral Nervous System after Injury. Biomedicines.

[19] Omura T., Omura K., Tedeschi A., Riva P., Painter M.W., Rojas L., Martin J., Lisi V., Huebner E.A., Latremoliere A. (2015). Robust Axonal Regeneration Occurs in the Injured CAST/Ei Mouse CNS. Neuron.

[20] Yu L., Yin Z., Huang R., Liu Z., Liu Y., Zheng X., Song S., Wang Z., He X., Bai Y. (2025). Activin A enhances neurofunctional recovery following traumatic spinal cord injury by inhibiting autophagy. Neural Regen. Res..

[21] Gudi V., Gai L., Herder V., Tejedor L.S., Kipp M., Amor S., Suhs K.W., Hansmann F., Beineke A., Baumgartner W. (2017). Synaptophysin Is a Reliable Marker for Axonal Damage. J. Neuropathol. Exp. Neurol..

[22] Kiya K., Kubo T. (2019). Neurovascular interactions in skin wound healing. Neurochem. Int..

[23] Malicevic U., Smith J., Agrawal D.K., Rai V. (2025). Sex-based differences in streptozotocin-induced type 2 diabetes rat models. J. Clin. Transl. Res..

[24] Cangkrama M., Wietecha M., Werner S. (2020). Wound Repair, Scar Formation, and Cancer: Converging on Activin. Trends Mol. Med..

[25] Antsiferova M., Werner S. (2012). The bright and the dark sides of activin in wound healing and cancer. J. Cell Sci..

[26] Li Y., Cheng Z., Yu F., Zhang Q., Yu S., Ding F., He Q. (2022). Activin A Secreted From Peripheral Nerve Fibroblasts Promotes Proliferation and Migration of Schwann Cells. Front. Mol. Neurosci..

[27] Andersen G.O., Ueland T., Knudsen E.C., Scholz H., Yndestad A., Sahraoui A., Smith C., Lekva T., Otterdal K., Halvorsen B. (2011). Activin A levels are associated with abnormal glucose regulation in patients with myocardial infarction: Potential counteracting effects of activin A on inflammation. Diabetes.

[28] Kuo C.S., Lu Y.W., Hsu C.Y., Chang C.C., Chou R.H., Liu L.K., Chen L.K., Huang P.H., Chen J.W., Lin S.J. (2018). Increased activin A levels in prediabetes and association with carotid intima-media thickness: A cross-sectional analysis from I-Lan Longitudinal Aging Study. Sci. Rep..

[29] Choi J.W., Nam K.M., Choi H.R., Huh C.H., Park K.C. (2018). Interactive Roles of Activin A in Epidermal Regeneration. Ann. Dermatol..

[30] Yaden B.C., Wang Y.X., Wilson J.M., Culver A.E., Milner A., Datta-Mannan A., Shetler P., Croy J.E., Dai G., Krishnan V. (2014). Inhibition of activin A ameliorates skeletal muscle injury and rescues contractile properties by inducing efficient remodeling in female mice. Am. J. Pathol..

[31] Latres E., Mastaitis J., Fury W., Miloscio L., Trejos J., Pangilinan J., Okamoto H., Cavino K., Na E., Papatheodorou A. (2017). Activin A more prominently regulates muscle mass in primates than does GDF8. Nat. Commun..

[32] Valtorta F., Pennuto M., Bonanomi D., Benfenati F. (2004). Synaptophysin: Leading actor or walk-on role in synaptic vesicle exocytosis?. Bioessays.

[33] Okajima S., Mizoguchi A., Masutani M., Tomatsuri M., Tamai K., Hirasawa Y., Ide C. (1993). Synaptophysin immunocytochemistry in the regenerating sprouts from the nodes of Ranvier in injured rat sciatic nerve. Brain Res..

[34] Liu M., Warn J.D., Fan Q., Smith P.G. (1999). Relationships between nerves and myofibroblasts during cutaneous wound healing in the developing rat. Cell Tissue Res..

[35] Zhao Y., Li Q., Jin A., Cui M., Liu X. (2015). E3 ubiquitin ligase Siah-1 downregulates synaptophysin expression under high glucose and hypoxia. Am. J. Transl. Res..

[36] Zhao L., Liu J.W., Kan B.H., Shi H.Y., Yang L.P., Liu X.Y. (2020). Acupuncture accelerates neural regeneration and synaptophysin production after neural stem cells transplantation in mice. World J. Stem Cells.

[37] Oliveira A.L., Thams S., Lidman O., Piehl F., Hokfelt T., Karre K., Linda H., Cullheim S. (2004). A role for MHC class I molecules in synaptic plasticity and regeneration of neurons after axotomy. Proc. Natl. Acad. Sci. USA.

[38] Kokotos A.C., Harper C.B., Marland J.R.K., Smillie K.J., Cousin M.A., Gordon S.L. (2019). Synaptophysin sustains presynaptic performance by preserving vesicular synaptobrevin-II levels. J. Neurochem..

[39] Tanner M.A., Grisanti L.A. (2021). A Dual Role for Death Receptor 5 in Regulating Cardiac Fibroblast Function. Front. Cardiovasc. Med..

[40] Lv Z., Hu J., Su H., Yu Q., Lang Y., Yang M., Fan X., Liu Y., Liu B., Zhao Y. (2025). TRAIL induces podocyte PANoptosis via death receptor 5 in diabetic kidney disease. Kidney Int..

[41] Steele H., Cheng J., Willicut A., Dell G., Breckenridge J., Culberson E., Ghastine A., Tardif V., Herro R. (2023). TNF superfamily control of tissue remodeling and fibrosis. Front. Immunol..

