# Supplementary material for: Hyperglycemia Impairs the Expression of Mediators of Axonal Regeneration During Diabetic Wound Healing in Rats
Source: Biomedicines. 2025 Dec 6;13(12):2994. doi: 10.3390/biomedicines13122994 (PMC12731212; doi:10.3390/biomedicines13122994)
Supplement: Supplementary file 1 [file biomedicines-13-02994-s001.zip › biomedicines-3966278-supplementary.pdf]

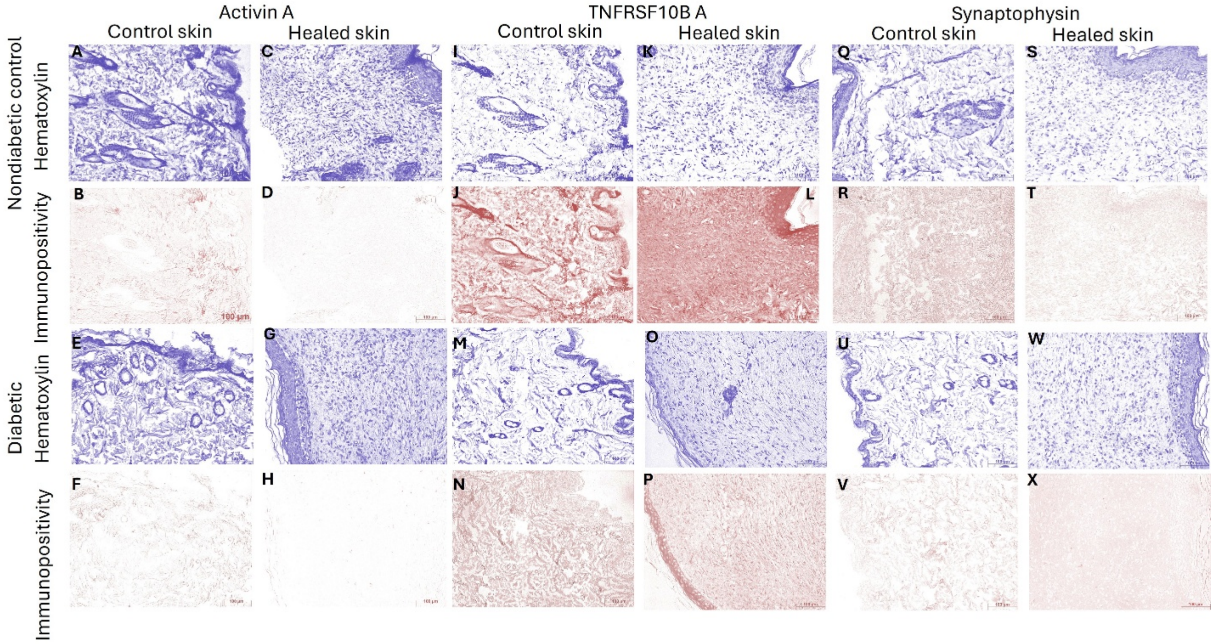

Supplementary Figure S1: Deconvulated images to show immunopositivity in different groups. Panels A-D, I-L, and Q-T (nondiabetic control rats) and panels E-H, M-P, and U-X (diabetic rats). Activin A (panels A-H), TNFRSF10B (panels I-P), and synaptophysin (panels Q-X). Control skin collected during wounding (panels A, B, E, F, I, J, M, N, Q, R, U, and V) and healed skin collected during sacrifice after the wound healed (panels (C, D, G, H, K, L, O, P, S, T, W, and X).
